# Peer Status Position within School-Based Hierarchies and Excessive Fat Accumulation in Adulthood—A 30 Year Follow up of a Stockholm Cohort

**DOI:** 10.3390/bs9080085

**Published:** 2019-08-09

**Authors:** Yerko Rojas, Ylva B. Almquist

**Affiliations:** 1 Swedish Institute for Social Research, Stockholm University, 10691 Stockholm, Sweden; 2 Social Sciences, Södertörn University, 14189 Huddinge, Sweden; 3 Department of Public Health Sciences, Stockholm University, 10691 Stockholm, Sweden

**Keywords:** body mass index (BMI), peer status, school, overweight, obesity, Sweden

## Abstract

Disadvantaged socioeconomic status is arguably the one exposure that has most consistently been linked to obesity, even more strongly so than diet and physical inactivity, which are the two main perceived root causes of weight gain. However, we still know very little about the relationship between having a disadvantaged social position and excessive fat accumulation, particularly when it comes to whether the relationship in question can also be seen as a long-term one, i.e., spanning from childhood to adulthood. By making use of the unique Stockholm Birth Cohort Multigenerational Study, the present study uses generalized ordered logistic regressions to examine the association between sociometrically assessed peer status position in school at age 13 and excessive fat accumulation at age 32. The results suggest that the odds of having excessive fat accumulation are about 0.5 times lower among popular and accepted children (ORs = 0.52 and 0.56, respectively), compared to those with a marginalized peer status position, independent of other obesogenic risk factors measured both prior and subsequent to peer status position. Our results give support to the notion that improved weight status may be another positive consequence of policies aiming to increase social inclusion within schools.

## 1. Introduction

Targeting social integration within schools, as a way of coming to terms with overweight and obesity among students [1], is being put forward as a potentially novel manner to approach a serious public health problem that has been proven difficult to solve with the dominant medically inspired perception of it, i.e., as a question reducible to genetics, diet, and sedentary lifestyle (cf. [2,3]). Previous studies have shown that low peer status positions within school-based hierarchies have a detrimental effect on excessive fat accumulation in adolescence, above and beyond other markers of social condition, such as parental education and family structure [4,5]. It has been suggested that lower social status in itself, through the processive stress that it may imply, may result in concurrent weight gain [4]. Although scholars have argued that we need to understand how this effect functions longitudinally [5], combined with the fact that there also is evidence relating obesity among adults to perceptions of low social status [6], we are unaware of any large-scale, prospective studies that have investigated whether a low peer status position within school-based hierarchies is associated with overweight and obesity in adulthood. This would seem to be an important research gap, not least from a prevention perspective.

Overall, little is known about whether the social mechanism associated with body mass status among adults pertains to childhood [6,7,8,9]. Part of the explanation for this limited knowledge is the lack of long-term follow-up data that span from childhood to adulthood [6,7,9]. By making use of the unique Stockholm Birth Cohort Multigenerational Study [10], the present study is able to explore the extent to which sociometrically assessed peer status position in school at age 13 is related to excessive fat accumulation at age 32, adjusting for well-established obesogenic risk factors measured both prior and subsequent to the measurement of low peer status position.

## 2. Materials and Methods

The data were derived from the Stockholm Birth Cohort Multigenerational Study (SBC Multigen) [10], which consists of 14,608 individuals (women: n = 7447; men: n = 7161) born in 1953 and registered as living in Sweden 1960, 1965, and/or 1968. The life situation of the cohort members has been repeatedly assessed over time using standardized interviews and surveys, as well as data available in administrative data registers. The main sources of information in the current study are: The School Survey of 1966; census data with 5-year intervals; obstetric data (1953); and the Culture and Leisure Time Survey of 1985. Ethical permission for the creation and use of SBC Multigen was obtained from The Regional Ethical Review Board in Stockholm (no. 2017/34-31/5; 2017/684-32).

The Culture and Leisure Time Study consisted of a mail-questionnaire distributed to a stratified sample of the cohort, with an additional oversampling of those who had scored in the highest 5% and lowest 5% on the cognitive ability test in the School Survey (resulting in a sample of approximately 30% of the cohort) (cf. [11]). Due to the exclusion of those with a lower BMI than normal i.e., underweight) at age 32, missing delivery records for those born outside Stockholm, and non-participation in the School Survey, the sample size for the final analysis in the model with full data for all the variables amounted to 1634 respondents (please see Table A1 in Appendix A for descriptive statistics).

For a more detailed description of SBC Multigen, including justification of its design, please see Almquist and colleagues [10].

### 2.1. BMI in Adulthood

Following WHO’s categorization based on body mass index (BMI), i.e., the weight in kilograms divided by the square of the height in meters (weight/height^2^), we define “Normal weight” as a BMI of 18.50–24.99, “Overweight” as a BMI of ≥25, and “Obesity” as a BMI of ≥30. BMI is a straight-forward and widely used measure that is easy to obtain and has furthermore been recommended for use in all age groups [12]. The cut-off points for overweight and obesity are based on public health and clinical considerations [13]. The measurements of weight and length stem from the Culture and Leisure Time Study.

### 2.2. Peer Status Position in the School Class

Peer status position was derived from the following question in the School Survey: “Which classmates do you best like to work with at school?”. The question includes dimensions of friendship, likeability and acceptance, but also, in part, school performance, (cf. [14]) and has been widely used in other studies (e.g., [15,16]). All students were asked to nominate three classmates. The number of received nominations was divided into four categories reflecting peer status position: “Marginalized” (0 nominations); “Peripheral” (1 nomination); “Accepted” (2–3 nominations); and “Popular” (4 or more nominations). These categorizations have been used previously (e.g., [17,18]). Given the focus of the current study, marginalized children serve as the reference group in our analyses.

### 2.3. Control Variables

Principal behavioral risk factors behind excessive fat accumulation [2,3] include sedentary lifestyle, tobacco habits (cf. [19,20]), and alcohol use (cf. [21]). In the current study, measures of these factors were derived from the Culture and Leisure Time Study. Sedentary behavior was defined through time spent watching television (TV) during an ordinary weekday and divided into three categories: “Approx. 3 or more hours”, “Approx. 1–2 h”, and “Approx. half an hour or less” (cf. [22]). Tobacco habits were defined through smoking and snus (smokeless tobacco products) habits, and categorized as: “Smoke”, “Snus only”, and “Do not smoke/snus”. Alcohol use was defined through a question about how much alcohol the individual needs to drink to become noticeably unsteady and was dichotomized in terms of: “Do not drink/have not drank enough to be affected” and “Other”.

Among the probable childhood predictors of adult excessive fat accumulation, and hence also plausible confounders of the association under study, recent reviews point to low parental socioeconomic status and high body weight, in particular birth weight [9,23]. Moreover, higher childhood IQ scores have been associated with a decreased prevalence of adult obesity [24]. In this study, parental socioeconomic status was measured through social class based on the father’s occupation in 1963 and divided into three categories: “Upper/upper middle class”, “Lower middle class”, and “Working class”. Birthweight was dichotomized as follows: “High birthweight” (having a birth weight above average, i.e., more than one standard deviation above the mean birthweight of the study population), and “Average or below average birthweight” (less than one standard deviation above the mean birthweight of the study population). Similarly, cognitive ability was defined in terms of “High cognitive ability” and “Average or below average cognitive ability”. This information was derived from a test on spatial, verbal, and numeric abilities included in the School Study.

Finally, and following previous research focusing on peer status position and BMI [5], gender (“Man” and “Woman”) and family type in 1964 (“Parents living together” and “Other”) were included as control variables.

### 2.4. Analytical Strategy

Under the assumption that there is a continuum running through the BMI categories of normal weight, overweight, and obesity [12,13], i.e., that these categories can be considered as part of a “group continuous” or ordered variable, the associations between peer status position in childhood and excessive fat accumulation in young adulthood were analyzed by means of generalized ordered logistic regression. This is a rather common form of modelling the BMI classification (cf. [25]).

The odds ratios (ORs) for the association between a given independent variable and excessive fat accumulation are to be interpreted as a proportional ORs, e.g., an OR of 3 would indicate that if the associated variable is increased by one, the odds of obesity versus the combined overweight and normal weight categories are 3 times greater, everything else held constant. Likewise, the odds of the combined overweight and obesity categories versus normal weight are 3 times greater, everything else held constant (please see Long and Freese [26] for a more detailed discussion, of how ORs are calculated in ordered logit models).

For the OR to be the same for each of the ordered dichotomizations of the outcome variable, the so-called parallel-line assumption, which is a characteristic of generalized ordered logit models, needs to hold [27,28]. The assessment of this precondition being met, as well as the actual analysis, was carried out by means of *gologit2* [27] in Stata [29]. Due to the stratified nature of the sample on which the Culture and Leisure Time Study is based, a specifically designed weight variable was taken into account by means of the *pweight* command in Stata (cf. [14])

The association between peer status position in school at age 13 (1966) and excessive fat accumulation at age 32 (1985) was, in a first step, analyzed in its crude form (Model 1). The association was subsequently adjusted for gender and adult health behaviors (sedentary lifestyle, tobacco habits, and alcohol use [1985]) (Model 2), childhood social conditions (family type [1964] and parental social class [1963]) (Model 3), and childhood individual characteristics (cognitive ability [1966] and birth weight [1953]) (Model 4).

## 3. Results

The results from the generalized ordered logit analysis are presented in Table 1. Model 1 shows that peer status position in school at age 13 is significantly related to overweight and obesity at age 32. The odds of having excessive fat accumulation were about 0.5 times smaller, both among popular and accepted children (ORs = 0.55 and 0.50, respectively), compared to those with a marginalized peer status position. As can be seen in Model 2, this relationship remains significant and practically the same after adjusting for gender and adult health behaviors (cf., OR = 0.57 with OR = 0.55 and OR = 0.51 with OR = 0.50, respectively). Of the introduced control variables, both gender and sedentary lifestyle are shown to be significantly related to overweight and obesity. Men have 2.4 times higher odds of having excessive fat accumulation than women (OR = 2.40). The odds of having excessive fat accumulation are about 1.5 times higher for those reporting watching TV 1–2 h on an ordinary weekday (OR = 1.50), compared to those who reported watching TV less than 1 h. The corresponding odds of having excessive fat accumulation for those watching TV 3 h or more are 2 times higher as compared to those who watched TV less than 1 h an ordinary weekday (OR = 2.00).

When family type and parental social class in childhood were included in the analysis, the odds of having excessive fat accumulation in adulthood remained significantly lower for those who had a popular (OR = 0.61) or accepted (OR = 0.53) peer status position in school, as compared to those who were marginalized. Of the introduced control variables, only parental social class was shown to be significantly related to overweight and obesity. The odds of having excessive fat accumulation were more than 2 times higher, both among those with working class and lower middle class parents (ORs = 2.56 and 2.19, respectively), compared to those with upper or upper middle class parents.

In the final model, birthweight and cognitive ability in childhood were introduced as additional control variables into the analysis. The odds of having excessive fat accumulation remained about 0.5 times lower, both among popular and accepted children (OR = 0.52 and 0.56, respectively), compared to those with a marginalized peer status position. Also gender, parental social class, and sedentary lifestyle remained statistically related to adult overweight and obesity.

As a way of examining how sensitive the final results were to the loss of cases (i.e., missing values) across the analytical models (i.e., Model 1:n = 2201, Model 2:n = 2130, Model 3:n = 2098, and Model 4:n = 1634), Models 1–3 were replicated including only the respondents in Model 4 (n = 1634). The effect of peer status position showed no sign of being affected by this. In other words, the results remained practically the same, both in terms of size and statistical significance throughout the models (data not shown).

## 4. Discussion

Overweight and obesity have been identified as the second leading cause of preventable disease and death in high-income countries (after smoking), and often referred to as one of the most important global health risk factors [30]. Furthermore, it is a problem that is increasing and becoming a major health concern also in the public mind [30,31,32]. Key international authorities have even described the situation as a global epidemic [33]. Sweden is not exempted from these trends [34]. In fact, more than a decade ago, the Swedish government set up a vision for 2021 of reducing the number of overweight and obese adults by at least 30% [35].

However, in order to address an epidemic, it is not only crucial to monitor prevalence and trends, and to identify effective strategies and intervention, but also to review its main determinants [7]. Disadvantaged socioeconomic status is arguably the one exposure that has most consistently been linked to obesity—even more strongly so than diet and physical inactivity, which are the two main perceived root causes of weight gain according to the medical model [3]. At a time when the traditional medically inspired perception of excessive fat accumulation is being proven unable to break the obesity epidemic [2,3], this is not to be taken lightly. Having said that, we still know very little about the relationship between disadvantaged social positions and excessive fat accumulation [3,36,37,38], and particularly when it comes to whether the relation in question can also be seen as a long-term one (cf. [6,9,23,37]).

Peer status position within schools is to be understood as a youth-specific indicator of social status. In fact, it was firstly introduced within the obesity literature as a novel and more accurate way of capturing this social dimension among children, as opposed to, for example, parental socioeconomic status [5]. The results of this study suggest that excessive fat accumulation in adulthood (age 32) can be traced back to low peer status position within school-based hierarchies (age 13), irrespective of gender, cognitive ability, and other obesogenic risk factors measured both prior (i.e., birthweight, family type, and parental social class) and subsequent (i.e., sedentary-lifestyle, tobacco habits, and alcohol use) to the measurement of low peer status position. This effect has previously been found for excessive fat accumulation in adolescence [4,5], but this is—to the best of our knowledge—the first time that this can also be said to hold true, in a large-scale, prospective sense, for overweight and obesity later in the life span.

A particular strength of this study is that we have used a sociometric measure to capture peer status position in the classroom—as opposed to a self-rated measure of social status position, which has been the main instrument used for this purpose within the literature [4,5,6]. Thus, our measurement is not afflicted by psychological biases, such as negative affect (cf. [39]). Moreover, it allows us to assert in a more objective manner the extent to which the respondents actually have been exposed to the stressful event of being in a marginalized position among classmates as children [16]. Finally, it allows us to include an adjacent stream of research into this research tradition. We know, for example, from earlier studies that popular children are less likely to exhibit learned helpless behavior (cf. [40]), which might very well be yet another pathway to adult obesity. Being limited in one’s ability to exercise control over one’s life has recently been put forward as key for understanding how low social status is related to excessive fat accumulation [36].

It is important to be cautious about drawing wide-ranging conclusions based on this single study. Nevertheless, our findings are most definitely a part of the growing collective evidence suggesting that improved weight status may be another positive implication of policies directed towards increasing social inclusion within schools (cf. [1]). Worth noticing, however, is that, although schools are a popular setting for intervention implementation given their continuous and intensive contact with children during their formative years, school-based programs addressing obesity specifically do not seem to have given this seemingly obesogenic interpersonal dimension within schools any type of consideration (cf. [41,42]). This might very well prove to be a rather ineffective strategy, since obesity preventions have been suggested to work better among individuals who feel in control and in good psychosocial health [36].

### Limitations

Several limitations should be born in mind when interpreting the results of this study. Using self-reported measures, in this case weight and height, may cause misclassification with regards to BMI (cf. [43]). Nonetheless, the research tradition on which this study is partly based on, commonly makes use of self-reported data (cf. [5]). In Sweden and elsewhere, monitoring and surveillance of overweight and obesity through self-reported data on height and weight is a well-established and widely used method (cf. [44,45]).

Moreover, we did not have access to data concerning the respondents body mass status at the time of the School Study. Hence, we are unable to completely rule out possible bias due to reverse causality, i.e., that children are not being chosen by their peers due to their weight (cf. [46]). On the other hand, the study does include a measure of the respondents’ weight prior to the measurement of sociometric status, namely, birthweight, which have been found to be predictive of obesity in childhood [47].

Given that diet plays such a central role in the medical model of obesity, it is a weakness that this study has not been able to control for whether the association between low peer status position in school and adult obesity exists above and beyond any given diet of the respondents. However, from a theoretical point of view, the mechanism through which stress, resulting from social position, increases visceral fat deposition is in part assumed to be a direct one, that is, independent of dietary intake (cf. [37]). Furthermore, a recent longitudinal study shows that the relation between low social status and obesity is strong and robust even when adjusted for dietary practices [6].

Other limitations of this study pertain to our measure of peer status position. Social status among youth is a complex phenomenon and our indicator captures only certain aspects (i.e., friendship, likability, and acceptance) in a specific context (i.e., the school), at a single time point. In young people’s everyday lives, status struggles take place along many other dimensions and in other settings. Status position is moreover a dynamic condition, which means that individuals may change their position in the status hierarchy over time. Nonetheless, given the robustness of findings from past research focusing on “snap shots” of peer status positions within school-based hierarchies (e.g., [14,16]), there are good reasons to believe that our measure is a viable indicator of social status.

Finally, this longitudinal study uses data from a Swedish 1953 birth cohort, with a follow-up spanning more than three decades. Perceptions of overweight and obesity are likely to have changed over time, as have the social arenas in which young people interact. There is, however, nothing to suggest that peer status would have become less important for later-life outcomes, including the processes leading up to excessive fat accumulation.

## 5. Conclusions

Excessive fat accumulation at age 32 is related to low peer status position in school at age 13. This impact of peer status position in childhood is independent of other consistent and robust obesogenic risk factors measured both at an earlier—e.g., birthweight—and later stage of the life span—e.g., sedentary lifestyle. Thus, policies directed towards reducing marginalization dynamics among peers within schools may be just as vital for targeting excessive fat accumulation as those that promote the improvement of nutrition education, healthy food choice, school meals, physical activity, and the like within schools. However, further studies are needed to confirm these findings.

## Figures and Tables

**Table 1:** Generalized ordered logistic regressions of BMI in adulthood and peer status position in childhood, Sweden, 1953–1983. Adjusted Odds Ratios (OR) with 95% confidence intervals (CIs).

	BMI in Adulthood (Ranging from Normal Weight, to Overweight, to Obesity)							
	Model 1		Model 2		Model 3		Model 4	
	OR	95% CI	OR	95% CI	OR	95% CI	OR	95% CI
**Peer status position**								
Popular	0.55	(0.36–0.85) *	0.57	(0.36–0.89) *	0.61	(0.39–0.95) *	0.52	(0.30–0.89) *
Accepted	0.50	(0.33–0.77) *	0.51	(0.33–0.80) *	0.53	(0.34–0.83) *	0.56	(0.33–0.94) *
Peripheral	0.81	(0.51–1.28)	0.81	(0.51–1.30)	0.79	(0.49–1.27)	0.84	(0.49–1.45)
Marginalized	1.00		1.00		1.00		1.00	
**Control variables**								
*Adult health behaviors*								
Sedentary lifestyle								
≥3 h TV a weekday			2.00	(1.20–3.34) *	1.72	(1.01–2.92) *	2.26	(1.25–4.07) *
1–2 h TV a weekday			1.45	(1.02–2.06) *	1.33	(0.93–1.91)	1.38	(0.91–2.12)
<1–0 h TV a weekday ^a^			1.00		1.00		1.00	
Tobacco habits								
Do not smoke/snus			1.14	(0.84–1.53)	1.18	(0.87–1.59)	1.07	(0.76–1.51)
Snus only			1.31	(0.82–2.08)	1.28	(0.78–2.08)	1.29	(0.73–2.31)
Smoke ^a^			1.00		1.00		1.00	
Alcohol use								
Don’t drink/haven’t drank enough to be affected			1.29	(0.91–1.83)	1.27	(0.89–1.80)	1.21	(0.81–1.81)
Other ^a^			1.00		1.00		1.00	
*Gender*								
Man			2.40	(1.76–3.29) *	2.38	(1.74–3.27) *	2.09	(1.47–2.97) *
Woman			1.00		1.00		1.00	
Family type								
Parents living together					0.83	(0.47–1.45)	0.90	(0.47–1.70)
Other ^a^					1.00		1.00	
*Socioeconomic position*								
Parental class								
Working class					2.56	(1.58–4.21) *	2.64	(1.46–4.78) *
Lower middle class					2.19	(1.34–3.58) *	2.31	(1.30–4.13) *
Upper/upper middle class ^a^					1.00		1.00	
*Mental test scores*								
Above average							1.26	(0.80–1.98)
Average and below ^a^							1.00	
*Birth weight*								
Above average							1.39	(0.93–2.08)
Average and below ^a^							1.00	
N	n = 2201		n = 2130		n = 2098		n = 1634	
Overweight	n = 268		n = 260		n = 254		n = 197	
Obesity	n = 44		n = 43		n = 43		n = 28	

^a^ Reference category; * Statistically significant (*p* < 0.05).

## Appendix A

**Table A1:** Distribution of dependent and independent variables included in respective models.

	Model 1 n = 2201 Percentages (%)		Model 2 n = 2130 Percentages (%)		Model 3 n = 2098 Percentages (%)		Model 4 n = 1634 Percentages (%)	
	Unweighted	Weighted	Unweighted	Weighted	Unweighted	Weighted	Unweighted	Weighted
**BMI in adulthood**								
Normal weight	85.8	86.3	85.8	86.2	85.8	86.2	86.2	86.6
Overweight	12.2	11.7	12.2	11.8	12.1	11.7	12.1	11.7
Obesity	2.0	2.0	2.0	2.1	2.1	2.1	1.7	1.7
**Peer status position**								
Popular	34.4	33.3	34.7	33.4	34.7	33.4	34.3	33.1
Accepted	36.6	36.7	36.2	36.2	36.2	36.2	37.1	37.1
Peripheral	18.0	18.7	18.1	18.8	18.8	18.0	17.6	18.5
Marginalized	11.0	11.4	11.1	11.5	11.2	11.6	11.1	11.3
**Control variables**								
*Adult health behaviours*								
Sedentary lifestyle								
≥ 3 h TV a weekday			8.5	8.3	8.4	8.2	8.7	8.3
1–2 h TV a weekday			63.7	66.2	63.6	66.2	62.2	66.1
<1–0 h TV a weekday ^a^			27.8	25.6	28.0	25.7	28.1	25.7
Tobacco habits								
Do not smoke/snus			48.9	47.1	49.2	47.3	50.1	48.3
Snus only			7.8	8.2	7.7	8.1	7.5	7.8
Smoke ^a^			43.3	44.8	43.1	44.6	42.4	43.9
Alcohol use								
Don’t drink/haven’t drank enough to be affected			23.9	23.9	24.0	24.0	23.7	23.6
Other ^a^			76.1	76.1	76.0	76.0	76.3	76.4
*Gender*								
Man			50.1	50.3	49.9	50.0	49.7	49.5
Woman			49.9	49.7	50.1	50.0	50.3	50.5
Family type								
Parents living together					94.3	94.0	94.3	93.8
Other ^a^					5.7	6.0	5.7	6.2
*Socioeconomic position*								
Parental class								
Working class					36.1	37.5	36.0	37.5
Lower middle class					43.9	45.0	44.1	45.4
Upper/upper middle class ^a^					20.0	17.5	19.9	17.1
*Mental test scores*								
Above average							30.5	17.5
Average and below ^a^							69.5	82.5
*Birth weight*								
Above average							15.4	15.5
Average and below ^a^							84.6	84.6
